# Coma Due to Acute Yellow Atrophy of the Liver

**Published:** 1910-11-26

**Authors:** 


### COMA DUE TO ACUTE YELLOW ATROPHY OF THE LIVER.

Coma due to acute yellow atrophy of the liver is usually associated with jaundice and preceded by convulsions. The disease is rare, but it is much commoner in women than in men. There is a close association between it and pregnancy. It has been known to follow a fright or great mental emotion.

In the majority of cases the illness begins with vomiting, epigastric pain, and jaundice, and is at first diagnosed as catarrhal jaundice. After a few days, however, the patient becomes much worse and complains of headache, tremor of the muscles, vomiting, hæmatemesis, delirium, and convulsions. Cutaneous hæmorrhages may be noticed, and if the patient is pregnant abortion occurs and is followed by coma. At this stage of the disease jaundice is usually well marked, the pulse is rapid, the liver dulness may be absent or much diminished, the spleen is enlarged, and the urine may be found to contain bile pigment, leucin, and tyrosin; a quantitative estimation of the urea will find it diminished to 1 per cent. or less.

The association of coma with jaundice, vomiting, diminution of the liver dulness, leucin and tyrosin in the urine, and a diminution in the amount of urea constitutes a group of symptoms which point in the most unmistakable manner to a diagnosis of acute yellow atrophy of the liver.

The following is a typical case:—

A woman aged twenty-six was admitted into hospital on December 22 on account of convulsions and coma, which had followed her sixth confinement. She had been married eight years, and had five children, one of whom died when six weeks old. Since her marriage she had worked at times as an office cleaner, but had never followed any occupation in which she had had to handle phosphorus. All the confinements with the exception of the last had been easy. On December 10 she first noticed pain in her back, and during the next few days she suffered from indigestion. On December 14 she gave birth to a girl. Unconsciousness followed, and on the 15th she suffered from epileptiform convulsions and vomiting. On the 18th jaundice was observed, and on the 22nd she was brought to the hospital in an unconscious condition. Her temperature was 97° F., pulse rate 140, and respiration rate 28 per minute. The breathing was short and of a sighing character. Soon after admission she vomited a curious mahogany-coloured viscid liquid, which was found to contain bile pigment, but no blood. She was insensitive to external impressions. The liver dulness was diminished, extending in the right nipple line only between the sixth and eighth ribs. There was marked jaundice. The urine had a specific gravity of 1019; it contained a good deal of bile pigment, 0.8 part of albumen per 1000, and only 1 per cent. of urea. Neither tyrosin nor leucin could be detected.

The blood was examined, with the following result:—

| | |
|---|---|
| Red blood corpuscles ... ... | 3,400,000 per cub. mm. |
| Hæmoglobin ... ... ... | 75 per cent. |
| White blood corpuscles ... | 31,875 per cub. mm. |

Differential Count of Leucocytes.

| | |
|---|---|
| Polymorphonuclear cells ... ... | 70 per cent. |
| Large lymphocytes ... ... ... | 7 „ |
| Small lymphocytes ... ... ... | 22 „ |
| Eosinophiles ... ... ... ... | 1 „ |

Leucin and tyrosin were both found in the blood. The patient rapidly sank, and died a few hours after admission.

At the autopsy the liver weighed 930 grammes (instead of 1,400 grammes). The capsule was loose and wrinkled, and the organ felt soft and flabby. It had the typical orange-brown rather than yellow blotchy look. On section it had a soft diffluent appearance. Sections examined under the microscope showed extensive necrosis of the hepatic cells.

